# Are taxes to sugar-sweetened beverages and non-essential energy dense food implemented in Mexico regressive?

**DOI:** 10.1371/journal.pone.0319922

**Published:** 2025-03-18

**Authors:** J. Alai Quiroz-Reyes, Jesús Enrique Morales-Ríos, Adriana Vargas-Flores, Néstor A. Sánchez-Ortiz, M. Arantxa Colchero

**Affiliations:** Center for Evaluation and Surveys Research, National Institute of Public Health, Cuernavaca, Morelos, México

## Abstract

**Introduction:**

In January 2014, Mexico introduced an excise tax of $1.00 Mexican peso/liter on sugar-sweetened beverages (SSB) and an 8% tax on non-essential energy dense food (NEDF) with at least 275 kilocalories/100 grams. Fiscal policies could be regressive when taxes generate a greater financial burden for low-income households compared to higher-income households. The objective of this study was to analyze whether SSB and NEDF taxes in Mexico were regressive using a nationally representative survey.

**Materials and methods:**

Information from the National Household Income and Expenditure Survey in Mexico in its 2014, 2016 and 2018 waves were used to estimate changes in expenditures on SSB and NEDF over total expenditures by income quintile and place of residence using own price elasticities, changes in prices after tax implementation and tax pass-through prices. We derived uncompensated own price elasticities using the Linear Approximation of the Almost Ideal Demand System.

**Results:**

Price elasticities -in absolute values- were higher in urban areas than in rural settings for SSB in 2014 and 2016, while the opposite was observed for NEDF. For SSB, price elasticities -in absolute values- among households in the highest income quintiles were lower than those in the lowest income quintiles. The tax paid for SSB and NEDF over total expenditures was higher among low-income households. However, the reduction in SSB expenditures over total expenditures in low-income households was higher compared to the highest income quintile. For NEDF, weekly expenditures in rural areas were lower than in urban areas for the lowest quintile, while for the highest quintile the reduction was similar.

**Conclusions:**

Low-income households in urban and rural areas reduced the proportion spent on SSB and NEDF more compared to higher-income residents, counteracting the regressive burden of the taxes.

## 1. Introduction

Sugar-sweetened beverages (SSB) and non-essential energy-dense food (NEDF) consumption in the world has increased significantly in the last decade [1,2]. In Mexico, SSB and NEDF combined represent 26% of the daily caloric intake [3,4]. SSB and NEDF consumption contribute to a higher caloric intake [5] and are associated with some diseases such as: obesity, malnutrition, hypertension, diabetes, among others [6–11].

The implementation of excise taxes has been effective in reducing the consumption of SSB in countries that implemented and evaluated their impact [12] and for NEDF in Mexico [13]. When the tax and tax passthrough rate are high, products may become less affordable and, thus, consumption and energy intake can decrease which may have an impact on obesity prevalence [14–18].

In January 2014, Mexico introduced an excise tax of $1.00 Mexican peso (MXN) per liter (L) on non-alcoholic SSB in powder, concentrate or ready to drink, energy drinks, with exception to medical products, 100% natural juices and beverages with artificial sweeteners [19]. The tax was adjusted when the cumulative inflation exceeded 10% increasing to $1.17 MXN in 2019. In January 2020, the tax became indexed to inflation annually [20] so the value in 2024 is $1.57 MXN per L [21]. In addition to the SSB tax, in January 2014, Mexico introduced a tax of 8% on NEDF that applies to snacks, confectionery products, chocolate and other products derived from cocoa, flans and puddings, fruit and vegetable candies, peanut and hazelnut creams, milk candies, cereal based foods, ice cream, and ice popsicles, with at least 275 kilocalories (kcal) per 100 grams (g) [19].

Fiscal policies could be regressive when taxes lead to a higher financial burden on low-income households compared to higher income ones [22]. Taxes can be regressive for low-income households as the amount spent on these products -as for any food or beverage- represent a higher proportion of their total expenditures compared to higher income households. Some studies show that SSB taxes are regressive because after tax implementation the proportion of SSB expenditures was higher among lower income household compared to those of higher income [23–28]. In contrast, a simulation study of a SSB tax in the United States found that higher-income households pay most of the tax [29]. An extended cost-benefit analysis that considers reductions of out-of-pocket expenditures associated with health gains, shows that by increasing SSB taxes, the effect on net income is progressive in the long term, with the lowest-income population being the most benefited compared to higher income households [30]. If lower income households experience a relatively higher own-price elasticity -in absolute terms- compared to higher income ones, a tax would reduce purchases to a larger extent and may reduce the proportion of SSB or NEDF over total expenditures more compared to high income households, thus, taxes would not be regressive. In low- and middle-income countries, poorer households spend a higher proportion of their income on SSB and NEDF and have higher price sensitiveness so taxes may not be regressive [31].

Most of the studies addressing this aspect of taxation come from the United States (US) and European countries, however, little evidence exists for middle- and low-income countries with high consumptions of SSB and NEDF [32]. As of our knowledge, no studies have been published to test if food and beverage taxes implemented in Mexico were regressive. The objective of this study was to analyze whether the SSB and NEDF taxes in Mexico were regressive using a nationally representative survey. Based on Fuchs’s methods [30], taxes are regressive if reductions in taxed food or beverages expenditures over total expenditures are larger among lower income households after tax implementation.

## 2. Materials and methods

### 2.1 Source of information

We used information from the National Household Income and Expenditure Survey in Mexico (ENIGH, for its Spanish acronym) in its 2014, 2016 and 2018 waves. The ENIGH is a biannual cross-sectional survey with a probabilistic, two-staged stratified clustered design, representative at national level and by area of residence (urban/rural). The survey collects information on food and beverage purchases for consumption at home (amount and expenditures) using a self-reported daily record instrument applied for a week. The ENIGH also includes information on household income and expenditures, sociodemographic characteristics of household members, occupation, housing infrastructure and household assets [33]*.* The ENIGH collects information of food and beverage purchases (quantity and amount paid) classified into 242 possible categories (that could be single or grouped foods). We sum expenditures by food or beverage group.

The data is publicly available with no identifiers at the household level (no names of household members, no location of the households) [33].

### 2.2 Food and beverage groups

For SSB we included soft drinks, juices and energy drinks. Although concentrates and syrups are taxed based on reconstitution, we excluded them for two reasons: we are unable to distinguished taxed versus untaxed and, as they are grouped together, we cannot apply a single formula for their reconstitution. For NEDF we included cookies, sweet bread by piece and packaged, pastries by piece and packaged, breakfast cereals, fried snacks, chips, processed fruit, chocolate bars, cajeta and sweets. We estimated total expenditures and quantity purchased for each group. We added a group of untaxed beverages that includes bottled water, milk and 100% juices.

### 2.3 Changes in SSB or NEDF expenditures

We first evaluated the regressive burden of the tax by estimating the amount of tax paid by income quintile and place of residence for SSB and NEDF. For SSB we multiplied the weekly quantity purchased of SSB in liters by the amount of the tax (1 peso/liter for 2014 and 2016, 1.1729 in 2018 as the tax was adjusted for inflation) over total household expenditures. Same estimations were conducted for NEDF but -as the tax is ad valorem- we multiplied the quantity purchased in kg by the tax paid (share of final retail price).

Then, to estimate changes in SSB and NEDF expenditures we followed the first part of the extended cost-benefit analysis methodology by Alan Fuchs *et al.* [30] which estimates the impact of taxes on household expenditures on SSB in Kazakhstan.

We estimated changes in expenditures by income quintile and place of residence for each wave of the ENIGH as follows:


$$\Delta Expenditure_{imjt}=\left[\left(1+\Delta P\right)\left(1+\varepsilon_{imj}*\Delta P_{m}\right)-1\right]*\frac{\omega_{imjt}}{Totalexpenditure_{mjt}}$$ (1)

where $\Delta Expenditure_{ij}$ corresponds to changes in expenditures per product *i*, place of residence *m*, income quintile *j* and wave *t*, $\Delta P_{m}$ corresponds to changes in prices by place of residence, $\varepsilon_{imj}$ is the price-elasticity per product *i* in place of residence *m* for each quintile *j* and $\omega_{imjt}$ is the proportion of expenditures in product *i* in place of residence *m* in wave *t* and income quintile *j*.

Changes in prices after tax implementation for SSB and NEDF for Mexico come from the literature. The tax pass-through prices for SSB and NEDF foods was heterogeneous. In urban areas, SSB prices increased by 1.08 pesos per liter, corresponding to 11% (100% pass-through) [34]. In rural areas, SSB prices increased by an average of 0.73 pesos per liter, corresponding to 8% (70% pass-through) [35]. For NEDF, prices increased 8% (100% pass-through) in urban areas [36], while in rural areas the price increased on average 4.5% (60% pass-through) [35]. The most recent meta-analysis shows a mean tax pass-through to prices of 80% [12]. We conducted a sensitivity analysis where we considered an 80% pass-through of the tax on prices.

### 2.4 Price elasticities

We estimated uncompensated own price elasticities for SSB and NEDF using the Linear Approximation of the Almost Ideal Demand System (LA/ AIDS) by Deaton and Muelbauer [37]. We modeled a demand system with three groups (SSB, NEDF and untaxed beverages that includes: bottled water, milk and 100% juices).

The LA/AIDS model includes Stone’s price index to obtain linear parameters. The Stone’s price index is constructed by multiplying the proportion of weekly expenditures for each product in the group by its price thus obtaining a weighted price for SSB, NEDF and untaxed beverages. Prices were derived by dividing expenditures by the quantity purchased for all foods and beverages that make up the SSB, NEDF and untaxed beverages groups. Finally, we added prices for all households at the municipal level to reduce the potential recall bias when households report expenditures and quantity purchased.

In each demand equation, the dependent variable is the proportion of household expenditures in product *i* over total expenditures in the system. The LA/AIDS system is given by:


$$W_{hmit}=\alpha_{i}+\overset{j}{\underset{j=1}{\sum }}\beta_{ij}logP_{mit}+\gamma_{ij}log\left(\frac{E}{P}\right)+\overset{k}{\underset{k=1}{\sum }}\delta_{ik}\eta_{htk}+\upsilon_{hmit}$$ (2)

h=1,…,H;m=1,…,M;i=1,…,j−1;t=2014,2016,2018

where $W_{hmit}$ is the proportion of expenditures for food or beverage group *i* for household *h* living in a municipality *m* during wave *t*; $P_{mit}$ is the price of group *i* at the municipal level *m* in wave *t*; *E* is the total household expenditure on SSB, NEDF and untaxed beverages, η are variables at the household and municipality level, and *log P* is the Stone’s price index. *h* and *m* represent the number of households and municipalities, respectively; while *k* denotes the number of covariates, these variables were: the sex of the head of the family (male/female), education of the head of the household in three categories: low for primary education, medium for secondary and high school and high for university or higher education. Quarterly household income was expressed in quintiles. Demand equations are constrained to fulfill the additivity $\overset{n}{\underset{i=1}{\sum }}\alpha_{i}=1$, $\overset{n}{\underset{i=1}{\sum }}\gamma_{i}=0$, $\overset{n}{\underset{i=1}{\sum }}\beta_{i}=0$, homogeneity $\overset{n}{\underset{i=1}{\sum }}\gamma_{ij}=0$ and symmetry $\gamma_{ij}=\gamma_{ji}$ restrictions.

We estimated the LA/AIDS model by income quintile and place of residence to get price elasticities for eachgroup. All analyses were stratified by survey wave to see if there were differences in price elasticities or in the proportion of SSB or NEDF expenditures over time.

All analyses were carried out with the STATA V.15 statistical package and consider the complex survey design of the ENIGH. We used the *quaids* command to estimate price elasticities and confidence intervals were derived by a clustered bootstrap with 300 repetitions.

## 3. Results

Table 1 shows descriptive statistics of the analytical sample by wave. About 72% of households reported spending on SSB, 76% on untaxed beverages and about 70% on NEDF. The distribution of household expenditures in the system was constant in all waves (about 38% in SSB, 26% in untaxed beverages, and 36% in NEDF). SSB price was around 0.82 USD per L in the three waves, the price of untaxed beverages decreased between 2014 and 2018, from 0.36 per L to 0.33 USD/L. The price of NEDF also decreased from 3.5 USD per kilogram (Kg) to 3.3 USD/Kg. Around 72% of the head of households were male in the three waves. For all waves, the largest proportion of households had medium education level and live in urban localities. Income in both urban and rural areas increased between waves, except for the highest income quintile in urban areas.

**Table 1:** Socioeconomic and demographic characteristics.

Characteristics	2014 (n = 17,812)	2016 (n = 64,383)	2018 (n = 68,190)
Percent of households with expenditures > 0			
Sugar-sweetened beverages	71.0%	73.2%	72.9%
Untaxed beverages	76.6%	75.4%	75.2%
Non-essential energy-dense food	68.0%	67.6%	66.8%
Distribution of household expenditures in the system			
SSB	34.6%	37.3%	37.5%
Untaxed beverages	26.4%	26.0%	26.1%
Non-essential energy-dense food	39.0%	36.7%	36.4%
Mean prices (USD 2023)			
Sugar-sweetened beverages (USD/liter)	0.82	0.81	0.82
Untaxed beverages (USD/liter)	0.36	0.34	0.33
Non-essential energy-dense food (USD/kilogram)	3.5	3.2	3.3
Head of the household sex (male)	75.0%	72.8%	72.0%
Education level (head of the household)			
Low (0–6 years of study)	39.9%	38.0%	36.2%
Medium (7–12 years of study)	43.9%	45.7%	46.5%
High (13 or more years of study)	16.2%	16.4%	17.3%
Urban localities ( > 2500 inhabitants)	79.2%	79.1%	77.8%
Household income by quintile (USD) and place of residence			
Urban			
Lowest	1,015.4	1,093.1	1,109.9
Low	1,804.6	1,934.3	1,957.9
Middle	2,619.3	2,779.4	2,809.5
High	3,891.8	4,049.1	4,077.9
Higher	9,451.5	9,344.3	9,120.0
Rural			
Lowest	566.0	663.6	655.3
Low	1,013.0	1,229.9	1.239.3
Middle	1,468.3	1,770.7	1,807.8
High	2,158.2	2,554.2	2,623.7
Highest	4,461.4	5,461.3	5559.3

Mean prices USD 2023. The complex survey design was considered in the estimations of mean and proportions. Source: own elaboration with information using the ENIGH [33].

Table 2 shows price elasticities by area of residence and income quintile. Price elasticities -in absolute values- were higher than one for SSB and NEDF for all households in all waves. Price elasticities -in absolute terms- were higher in rural areas than in urban settings for SSB in all waves: with the same price increase, consumption in rural areas would decrease more compared to urban areas. The same was observed for NEDF in 2018. For SSB, price elasticities -in absolute values- were lower among households in the highest income quintiles compared to those in the lowest quintiles.

**Table 2:** Uncompensated price elasticities by place of residence and income quintile.

	2014		2016		2018	
	Rural	Urban	Rural	Urban	Rural	Urban
**Sugar-sweetened beverages**						
Total	−1.10	−1.0	−1.15	−1.03	−1.25	−1.08
	(−1.24, −0.97)	(−1.06, −0.92)	(−1.20, −1.10)	(−1.08, −0.98)	(−1.33, −1.21)	(−1.14, −1.02)
Lowest	−1.30	−1.18	−1.20	−1.18	−1.43	−1.31
	(−1.56, −1.08)	(−1.29, −1.07)	(−1.30, −1.12)	(−1.27, −1.09)	(−1.56, −1.32)	(−1.39, −1.20)
Low	−1.10	−0.97	−1.18	−1.02	−1.38	−1.11
	(−1.40, −0.82)	(−1.13, −0.81)	(−1.31, −1.07)	(−1.11, −0.90)	(−1.48, −1.26)	(−1.22, −0.98)
Middle	−1.14	−0.96	−1.05	−1.02	−1.14	−1.02
	(−1.36, −0.91)	(−1.11, −0−83)	(−1.17, −0.94)	(−1.11, −0.94)	(−1.25, −1.01)	(−1.14, −0.89)
High	−1.02	−0.93	−1.03	−0.92	−1.05	−0.88
	(−133, −0.67)	(−1.09, −0.78)	(−1.13, −0.90)	(−1.01, −0.80)	(−1.18, −0.93)	(−0.98, −0.78)
Highest	−0.91	−0.75	−1.11	−0.92	−0.98	−0.83
	(−1.16, −0.66)	(−0.88, −0.63)	(−1.23, −0.98)	(−1.00, −0.84)	(−1.12, 0.86)	(−0.97, −0.71)
**Non-essential energy-dense food**						
Total	−1.07	−1.0	−1.11	−1.11	−1.29	−1.17
	(−1.22, −0.92)	(−1.06, −0.92)	(−1.15, −1.07)	(−1.15, −1.08)	(−1.37, −1.25)	(−1.21, −1.12)
Lowest	−1.00	−1.11	−1.01	−1.12	−1.36	−1.23
	(−1.32, −0.74)	(−1.25, −0.98)	(−1.14, −0.91)	(−1.20, −1.05)	(−1.53, −1.21)	(−1.35, −1.09)
Low	−1.00	−1.01	−1.00	−1.13	−1.29	−1.16
	(−1.31, −0.65)	(−1.14, −0.82)	(−1.09, −0.90)	(−1.21, −1.05)	(−1.41, −1.16)	(−1.26, −1.02)
Middle	−1.11	−0.95	−1.12	−1.10	−1.23	−1.18
	(−1.36, −0.85)	(−1.08, −0.78)	(−1.25, −1.03)	(−1.18, −1.02)	(−1.36, −1.09)	(−1.28, −1.06)
High	−1.14	−0.99	−1.08	−1.06	−1.19	−1.03
	(−1.53, −0.84)	(−1.13, −0.85)	(−1.16, −1.00)	(−1.14, −0.98)	(−1.34, −1.06)	(−1.14, −0.91)
Highest	−1.14	−0.83	−1.24	−1.09	−1.13	−1.03
	(−1.43, −0.87)	(−0.96, −0.68)	(−1.36, −1.13)	(−1.17, −0.99)	(−1.27, −1.02)	(−1.20, −0.93)

Confidence intervals in parentheses. Source: own elaboration using the National Income and Expenditure Surveys (ENIGH) [33].

For SSB in rural areas the average price elasticity of demand in 2014, 2016 and 2018 was −1.1, −1.15, and −1.25, respectively, which implies that a 10% price increase would result in reduction in the quantity purchased of 11%, 11.5% and 12.5%, respectively. In urban areas, and 10% increase in price is associated with a reduction in the quantity purchased of 10% in 2014, 10.3% in 2016 and 10.8% in 2018. For NEDF, the average price elasticity in rural areas was −1.07 in 2014, −1.11 in 2016 and −1.29 in 2018; in urban areas −1.0, −1.11 and −1.17, in 2014, 2016 and 2018, respectively.

In 2018, for both SSB and NEDF, households in the lowest income quintile had a higher price elasticity -in absolute value- compared to those in the highest quintiles, both in rural and urban areas. The same gradient is observed in 2014 and 2016 for SSB, but not for NEDF.

Table 3 shows the proportion of SSB or NEDF weekly expenditures over total expenditures. In general, households in the lowest quintiles in rural areas are those that allocate a greater proportion of their weekly spending on these products compared to higher income quintiles. The proportion of SSB and NEDF over total expenditures in rural areas is higher compared to urban areas in all waves.

**Table 3:** Proportion SSB and NEDF over total weekly expenditures by place of residence and income quintile.

	2014		2016		2018	
	Rural	Urban	Rural	Urban	Rural	Urban
**Sugar-sweetened beverages**						
Lowest	6.5%	4.5%	5.9%	4.3%	6.3%	4.3%
Low	5.0%	3.4%	4.5%	3.4%	4.9%	3.3%
Middle	4.1%	3.1%	3.9%	3.1%	3.9%	2.8%
High	3.8%	2.8%	3.6%	2.5%	3.3%	2.4%
Highest	3.0%	1.7%	2.7%	1.7%	2.6%	1.6%
**Non-essential energy-dense food**						
Lowest	4.6%	3.5%	4.5%	3.4%	4.4%	3.5%
Low	4.1%	2.8%	3.5%	2.8%	3.2%	2.8%
Middle	3.1%	2.7%	3.1%	2.4%	2.9%	2.4%
High	2.7%	2.2%	2.8%	2.2%	2.3%	2.1%
Highest	2.4%	1.5%	2.2%	1.6%	1.9%	1.5%

Source: own elaboration using the National Income and Expenditure Surveys (ENIGH) [33].

Table 4 shows changes in weekly expenditures on SSBs and NEDF in rural and urban areas and by income quintile. Households in urban areas and those belonging to the lowest quintiles decreased their weekly expenditures on these products more compared to higher income households.

**Table 4:** Change in SSB and NEDF expenditures over total expenditures by income quintile and place of residence.

	2014		2016		2018	
	Rural	Urban	Rural	Urban	Rural	Urban
**Sugar-sweetened beverages**						
Lowest	−0.18%	−0.15%	−0.13%	−0.13%	−0.25%	−0.22%
	(−019%, −0.16%)	(−0.16%, −0.14%)	(−0.14%, −0.13%)	(−0.13%, −0.14%)	(−0.26%, −0.24%)	(−0.23%, −0.21%)
Low	−0.13%	−0.04%	−0.09%	−0.07%	−0.16%	−0.10%
	(−0.15%, −0.12%)	(−0.05%, −0.04%)	(−0.09%, −0.08%)	(−0.07%, −0.06%)	(−0.17%, −0.15%)	(−0.10%, −0.09%)
Middle	−0.04%	−0.02%	−0.05%	−0.04%	−0.08%	−0.04%
	(−0.05%, −0.04%)	(−0.01%, −0.02%)	(−0.05%, −0.05%)	(−0.05%, −0.04%)	(−0.08%, −0.08%)	(−0.04%, −0.04%)
High	−0.01%	−0.003%	−0.03%	−0.02%	−0.04%	−0.007%
	(−0.02%, −0.01%)	(−0.004%, −0.002%)	(−0.03%, −0.03%)	(−0.03%, −0.02%)	(−0.04%, −0.03%)	(−0.007%, −0.006%)
Highest	0.01%	0.002%	−0.01%	−0.01%	0.004%	−0.008%
	(0.004%, 0.009%)	(0.002%, 0.003%)	(−0.01%, −0.01%)	(−0.01%, −0.01%)	(0.003%, 0.004%)	(−0.008%, −0.008%)
**Non-essential energy-dense food**						
Lowest	−0.02%	−0.05%	−0.02%	−0.04%	−0.07%	−0.11%
	(−0.03%, −0.02%)	(−0.05%, −0.04%)	(−0.02%, −0.02%)	(−0.05%, −0.04%)	(−0.07%, −0.07%)	(−0.11%, −0.10%)
Low	−0.02%	−0.03%	−0.02%	−0.04%	−0.05%	−0.07%
	(−0.02%, −0.02%)	(−0.03%, −0.03%)	(−0.02%, −0.02%)	(−0.05%, −0.04%)	(−0.05%, −0.05%)	(−0.07%, −0.07%)
Middle	−0.01%	−0.02%	−0.02%	−0.04%	−0.04%	−0.05%
	(−0.01%, −0.009%)	(−0.03%, −0.02%)	(−0.02%, −0.02%)	(−0.04%, −0.04%)	(−0.04%, −0.03%)	(−0.05%, −0.05%)
High	−0.008%	−0.02%	−0.02%	−0.03%	−0.03%	−0.03%
	(−0.008%, −0.007%)	(−0.02%, −0.01%)	(−0.02%, −0.02%)	(−0.03%, −0.03%)	(−0.03%, −0.02%)	(−0.03%, −0.03%)
Highest	−0.004%	−0.006%	−0.01%	−0.03%	−0.01%	−0.02%
	(−0.005%, −0.003%)	(−0.007%, −0.006%)	(−0.01%, −0.01%)	(−0.03%, −0.02%)	(−0.01%, −0.01%)	(−0.02%,−0.02%)

Source: own elaboration using the National Income and Expenditure Surveys (ENIGH) [33].

In 2014, the reduction in weekly expenditures on SSBs was -0.18% in rural areas and -0.15% in urban areas for the lowest income quintile, while for the highest income households there was an increase 0.01% and 0.002% respectively. For 2016, SSB the reduction is the same in both rural and urban areas (−0.13%) for the lowest income quintile, while for the highest quintile it was −0.01% in both areas. In 2018 the reduction in SSBs spending was −0.25% in rural areas and −0.22 in urban areas in the lowest quintile and there was an increase 0.004% in rural areas and reduction −0.008% in urban areas in the highest quintile.

For NEDF in 2014, weekly spending had a reduction of −0.02% in rural areas and −0.05% in urban areas in the lowest quintile, and −0.004% and −0.006% in the highest income quintile. In both 2016 and 2018, greater reductions are observed in urban areas (−0.04% and −0.11% respectively) than in rural areas (−0.02% and −0.07%) in the lowest income quintile. For the highest income quintile, the reduction was −0.01 in 2016 and 2018 in rural areas, while for urban areas it was −0.03 and −0.02 respectively. Overall, the reductions in changes of weekly spending were greater for SSBs than for non-essential energy-dense foods.

Although the lowest income quintiles purchased lower amounts of SSB and NEDF, the amount paid of taxes over total expenditures was higher as their income is lower compared to higher income quintiles (S1, S2 and S3 Tables). Compared to urban areas, the tax burden is higher for households in rural areas (S1, S2 and S3 Tables). However, as shown in Table 4, reductions in SSB and NEDF expenditures over total expenditures were higher among the lowest income households, counteracting the regressive burden of the taxes.

Results from the sensitivity analysis to pass-through show that households in the poorest quintiles reduce both SSB and NEDF expenditure more over total expenditure compared to households in the highest income quintiles (S4 Table).

## 4. Discussion

We evaluated the potential regressivity of SSB and NEDF taxes implemented in Mexico by estimating changes in the proportion of SSB and NEDF expenditures over total expenditures after the taxes were implemented. We found that lower income households in urban and rural areas reduced more the proportion spent on SSB and NEDF compared to higher income dwellers implying that the taxes were not regressive. This is expected for SSB as price elasticities -in absolute values- are higher for lower income and because, although the proportion purchased over expenditures is higher among lower income households, the quantity purchased is lower compared to highest income households (S1, S2 and S3 Tables). For NEDF, prices elasticities and quantity purchased are similar across income quintiles.

Price elasticities estimated in our study are in the range of those estimated for Mexico and similar countries. Andreyeva et al. (2022) that conducted a meta-analysis to evaluate the effectiveness of SSB taxes in high− and middle−income countries, estimated an average price elasticity of −1.59 (95% C.I., −2.11 to −1.08) and between −2.0 and −0.29 for Mexico (−0.78 average) [12] , while another study for Mexico estimates a price elasticity of − 0.951 [38]. In countries with similar characteristics such as Chile the price elasticity was estimated at −1.56 [12], South Africa (−1.55) [39], Guatemala (−1.39 for soft drinks) [40], Malaysia (−1.11) [41], Brazil (−0.85) [42], and Barbados (−0.73) [12].

For NEDF, we estimated on average a price elasticity of −1.18, which in absolute terms is higher than that for sweets/candies (0.34) shown by Andreyeva et al. (2010) [43]. Venson et al. (2023) estimated higher elasticities in Brazil compared Mexico, for ice cream the elasticity is −1.81, snacks and pizza −3.49, pastries −3.44 and a positive elasticity in sweets (0.11) [44]. In Chile, a price elasticity was estimated at −1.95 for salty snacks and chips, −1.30 [45].

The higher reductions in the proportion of SSB and NEDF over total expenditures in urban compared to rural areas after the taxes were implemented is expected as price elasticities and the tax pass-through were higher in urban settings [34–36]. Our results showing that SSB price elasticities in rural areas are higher -in absolute values- than in urban areas are consistent with a study in Guatemala showing a price elasticity of the demand for SSB of −2.09 in rural areas and −0.8 in urban areas [40]. A report from Indonesia, shows higher price elasticities in rural than in urban areas [46].

We estimated price elasticities using a LA/AIDS model. We selected a LA/AIDS over QUAIDS because AIDS models provide more precise estimates as they produce lower mean squared errors and mean absolute relative errors compared to QUAIDS in price elasticity estimations [47]. In addition, LA/AIDS models showed the expected gradient in price elasticities across income quintiles [41]. For SSB, price elasticities are in line with results from other studies [48–50]. Moreover, SSB price elasticities in the highest income quintiles were lower -in absolute values- than those for lowest income household which is consistent with the literature [30].

Results of the study show that the SSB and NEDF taxes implemented in Mexico were not regressive. In other countries where reductions in expenditures of a taxed product over total expenditures among the poor are not higher compared to the richer after the tax is implemented, if benefits on health (reductions in health expenditures) are higher among the poor, taxes may not regressive [30]. For our study we did not estimate the benefits on health as we showed that although taxes paid represented a higher burden for the poorest, reductions in SSB and NEDF expenditures over total expenditures after tax implementation were larger among lower income households, counteracting the regressive burden of the taxes. However, if there is a positive effect on health, the poorest would benefit more as their access to health care services is limited -particularly for the population working in the informal sector that lacks health insurance [51,52]- and their out-of-pocket expenses as a percentage of income is higher [53]. A recent study predicts that an increase in the SSB tax would benefit more low- and middle-income households with the highest reductions in obesity -the lowest income quintile would get slightly lower reductions in obesity as their baseline rates are lower [54].

Our study has some limitations. Self-reported information may be biased, food and beverage purchased may be underestimated but there is no reason to believe that there is a differential bias over time. We acknowledge that prices derived from expenditures and quantity purchased for each good may be biased due to recall biases. To reduce this potential bias, we used aggregated prices at the municipal level. Prices could also be endogenous if individuals have more information/skills to find optimal prices as described by Zhen (2014) [28] who used a weighted average price index using distances between households as an instrumental variable. Unfortunately, for confidentiality, ENIGH does not provide the geolocation of households. We also recognize that we used household expenditures on food and beverages consumed at home as the survey does not provide the specific goods consumed away from home which accounts for about 20% of total expenditures. SSB and NEDF purchases could be underestimated since they are based on a self-reported survey, but this would not change after the taxes were implemented. We acknowledge that taxed beverages include untaxed soft drinks but according to Euromonitor 2022, taxed soft drinks represent 76% of total taxed beverages and untaxed soft drinks represent 3.6% of all beverages excluding bottled water [55]. Although we excluded concentrates and syrups, they represented less than 4% of SSB expenditures as for 2018. We acknowledge that our analysis failed to differentiate changes in purchases associated with the tax from changes in tastes or demographics over time as we lack a comparison group. However, the period of analysis is short (four years) to see significant changes in these variables over time.

Despite the limitations, as of our knowledge, this is the first study that analyzes the potential regressivity of SSB and NEFD taxes in Mexico. The SSB tax is low as it represents 6.5% of final price, much lower than other countries in the region with SSB taxes such as Belize, Chile, Ecuador [56]. Knowing that SSB and NEDF are not regressive, a fiscal reform to increase both taxes would not represent a higher burden for the poor.

## Supporting information

S1 Table Weekly expenditures by income quintile and place of residence. (PDF)

S2 Table Quantity of SSB or NEDF purchased weekly by income quintile and place of residence. (PDF)

S3 Table Proportion of tax paid over weekly expenditures. (PDF)

S4 Table Changes in weekly expenditures by income quintile and place of residence. Sensitivity analysis to pass through prices.
(PDF)
