# Supplementary material for: Are taxes to sugar-sweetened beverages and non-essential energy dense food implemented in Mexico regressive?
Source: PLoS One. 2025 Mar 18;20(3):e0319922. doi: 10.1371/journal.pone.0319922 (PMC11918417; doi:10.1371/journal.pone.0319922)
Supplement: S1 Table — (PDF) [file pone.0319922.s001.pdf]

S1 Table- Weekly expenditures by income quintile and place of residence

	ENIGH 2014			ENIGH 2016			ENIGH 2018		
	Rural	Urban	Total	Rural	Urban	Total	Rural	Urban	Total
**SSB**									
Lowest	58.15	90.32	76.34	64.95	93.52	80.13	64.43	94.64	107.50
Low	77.36	128.71	111.96	90.26	129.32	112.03	88.65	131.53	113.30
Middle	98.01	158.93	143.00	112.30	165.68	84.71	115.30	172.18	150.57
High	131.07	218.78	195.58	142.10	220.00	195.75	151.33	226.26	200.72
Highest	215.20	452.90	410.33	234.45	399.66	360.39	248.19	420.85	375.39
**Non-essential energy-dense food**									
Lowest	55.56	88.14	72.47	60.46	91.50	75.66	60.68	92.95	75.81
Low	74.82	127.41	112.19	88.48	130.52	112.58	148.96	132.04	113.44
Middle	98.67	161.49	144.83	112.86	168.27	148.27	116.06	175.12	151.68
High	135.20	223.07	199.27	141.89	223.67	198.43	149.59	230.39	204.41
Highest	221.07	470.50	424.21	238.98	426.93	382.19	252.03	441.34	391.25

Expenditures in USD 2023. Own elaboration using information from ENIGH.
