# Supplementary material for: Are taxes to sugar-sweetened beverages and non-essential energy dense food implemented in Mexico regressive?
Source: PLoS One. 2025 Mar 18;20(3):e0319922. doi: 10.1371/journal.pone.0319922 (PMC11918417; doi:10.1371/journal.pone.0319922)
Supplement: S2 Table — (PDF) [file pone.0319922.s002.pdf]

S2 Table- Quantity of SSB or NEDF purchased weekly by income quintile and place of residence

	ENIGH 2014			ENIGH 2016			ENIGH 2018		
	Rural	Urban	Total	Rural	Urban	Total	Rural	Urban	Total
**SSB**									
Lowest	3.71	3.88	3.75	3.77	3.86	3.81	3.73	3.81	3.76
Low	3.75	4.60	4.47	4.19	4.54	4.26	4.20	4.49	4.29
Middle	4.62	5.30	5.00	4.43	5.25	4.98	4.60	4.97	4.80
High	4.96	5.99	5.72	5.14	5.66	5.54	5.01	5.47	5.30
Highest	5.40	5.86	5.91	5.96	5.77	5.81	5.90	5.60	5.66
**Non-essential energy-dense food**									
Lowest	1.03	1.07	1.08	1.12	1.06	1.08	1.00	0.99	0.99
Low	0.99	1.01	1.03	1.27	1.13	1.18	1.15	1.10	1.09
Middle	0.99	1.14	1.07	1.28	1.22	1.25	1.24	1.16	1.17
High	1.24	1.26	1.25	1.51	1.23	1.22	1.28	1.20	1.20
Highest	1.43	1.24	1.26	1.40	1.45	1.42	1.44	1.32	1.33

SSB in Liters. NEDF in Kilograms. Own elaboration using information from ENIGH.
