# Supplementary material for: Are taxes to sugar-sweetened beverages and non-essential energy dense food implemented in Mexico regressive?
Source: PLoS One. 2025 Mar 18;20(3):e0319922. doi: 10.1371/journal.pone.0319922 (PMC11918417; doi:10.1371/journal.pone.0319922)
Supplement: S3 Table — (PDF) [file pone.0319922.s003.pdf]

S3 Table- Proportion of tax paid over weekly expenditures

	ENIGH 2014			ENIGH 2016			ENIGH 2018		
	Rural	Urban	Total	Rural	Urban	Total	Rural	Urban	Total
**SSB**									
Lowest	0.36%	0.24%	0.28%	0.33%	0.23%	0.27%	0.38%	0.27%	0.23%
Low	0.27%	0.20%	0.23%	0.26%	0.20%	0.21%	0.31%	0.23%	0.25%
Middle	0.27%	0.19%	0.20%	0.22%	0.18%	0.33%	0.26%	0.19%	0.21%
High	0.21%	0.15%	0.16%	0.20%	0.15%	0.16%	0.22%	0.16%	0.17%
Highest	0.14%	0.07%	0.08%	0.14%	0.08%	0.09%	0.16%	0.09%	0.10%
**Non-essential energy-dense food**									
Lowest	0.44%	0.29%	0.35%	0.40%	0.25%	0.31%	0.37%	0.24%	0.29%
Low	0.31%	0.19%	0.22%	0.31%	0.19%	0.23%	0.17%	0.19%	0.21%
Middle	0.24%	0.17%	0.18%	0.25%	0.16%	0.18%	0.24%	0.15%	0.17%
High	0.22%	0.13%	0.15%	0.23%	0.12%	0.13%	0.19%	0.12%	0.13%
Highest	0.15%	0.06%	0.07%	0.13%	0.07%	0.08%	0.13%	0.07%	0.08%

Own elaboration using information from ENIGH.
