# Supplementary material for: Are taxes to sugar-sweetened beverages and non-essential energy dense food implemented in Mexico regressive?
Source: PLoS One. 2025 Mar 18;20(3):e0319922. doi: 10.1371/journal.pone.0319922 (PMC11918417; doi:10.1371/journal.pone.0319922)
Supplement: S4 Table — Sensitivity analysis to pass through prices. (PDF) [file pone.0319922.s004.pdf]

S4 Table. Changes in weekly expenditures by income quintile and place of residence
Sensitivity analysis to pass through prices

	ENIGH 2014		ENIGH 2016		ENIGH 2018	
	Rural	Urban	Rural	Urban	Rural	Urban
**SSB**						
Lowest	-0.13%	-0.11%	-0.10%	-0.10%	-0.19%	-0.18%
	(-0.14%, -0.12%)	(-0.12%, -0.10%)	(-0.10%, -0.10%)	(-0.10%, -0.09%)	(-0.20%, -0.18%)	(-0.17%, -0.16%)
Low	-0.10%	-0.03%	-0.06%	-0.05%	-0.12%	-0.07%
	(-0.11%, -0.09%)	(-0.03%, -0.03%)	(-0.07%, -0.06%)	(-0.05%, -0.04%)	(-0.13%, -0.12%)	(-0.07%, -0.07%)
Middle	-0.03%	-0.008%	-0.04%	-0.03%	-0.06%	-0.03%
	(-0.03%, -0.03%)	(-0.008%, -0.008%)	(-0.04%, -0.03%)	(-0.03%, -0.03%)	(-0.06%, -0.06%)	(-0.03%, -0.03%)
High	-0.01%	-0.002%	-0.02%	-0.01%	-0.02%	-0.001%
	(-0.008%, -0.006%)	(-0.001%, -0.003%)	(-0.02%, -0.02%)	(-0.02%, -0.01%)	(-0.03%, -0.02%)	(-0.001%, -0.001%)
Highest	0.008%	0.02%	-0.006%	-0.005%	0.005%	0.009%
	(0.006%,0.01%)	(0.002%,0.002%)	(-0.006%, -0.006%)	(-0.005%, -0.005%)	(0.005%,0.005%)	(0.009%,0.009%)
**Non-essential energy-dense food**						
Lowest	-0.02%	-0.03%	-0.01%	-0.03%	-0.05%	-0.08%
	(-0.02%, -0.02%)	(-0.03%, -0.03%)	(-0.01%, -0.01%)	(-0.03%, -0.03%)	(-0.05%, -0.05%)	(-0.09%, -0.08%)
Low	-0.02%	-0.02%	-0.01%	-0.03%	-0.04%	-0.05%
	(-0.02%, -0.02%)	(-0.02%, -0.02%)	(-0.01%, -0.01%)	(-0.03%, -0.03%)	(-0.04%, -0.04%)	(-0.06%, -0.05%)
Middle	-0.007%	-0.02%	-0.01%	-0.03%	-0.03%	-0.04%
	(-0.007%, -0.007%)	(-0.02%, -0.02%)	(-0.01%, -0.01%)	(-0.03%, -0.03%)	(-0.03%, -0.03%)	(-0.04%, -0.04%)
High	-0.001%	-0.020%	-0.01%	-0.02%	-0.02%	-0.02%
	(-0.001%, -0.001%)	(-0.01%, -0.009%)	(-0.01%, -0.01%)	(-0.02%, -0.02%)	(-0.02%, -0.02%)	(-0.02%, -0.02%)
Highest	0.0%	0.006%	-0.01%	-0.02%	-0.007%	-0.01%
	(0.00%,0.00%)	(0.006%,0.007%)	(-0.01%, -0.009%)	(-0.02%, -0.02%)	(-0.007%, -0.007%)	(-0.01%, -0.01%)

Source: own elaboration using information from ENIGH.
